# Comparing the Effects of Contact-Based Education and Acceptance and Commitment-Based Training on Empathy toward Mental Illnesses among Nursing Students 

**Published:** 2018-04

**Authors:** Saeed Vaghee, Masoud Kashani Lotfabadi, Azam Salarhaji, Nastaran Vaghei, Bibi Maryam Hashemi

**Affiliations:** 1 Psychiatry Faculty, University of Social Welfare and Rehabilitation Sciences, Tehran, Iran.; 2 Department of Psychiatry, Tehran University of Medical Sciences, Tehran, Iran.; 3 Research Center for Cognitive and Behavioral Sciences, Tehran University of Medical Sciences, Tehran, Iran.; 4 Psychiatry and Psychology Research Center, Tehran University of Medical Sciences, Tehran, Iran.; 5 Department of Epidemiology and Biostatistics, School of Public Health, Tehran University of Medical Sciences, Tehran, Iran.

**Keywords:** *Acceptance and Commitment Therapy*, *Education*, *Mental Disorders*, *Nursing*, *Empathy*, *Students*

## Abstract

**Objective:** Empathy is an important and valuable tool in therapeutic communication. Improvement barriers of empathy in psychiatric nursing education are associated with challenges, such as stress due to negative attitudes toward psychiatric disorders. The current study aimed at comparing the effects of contact-based education and commitment and acceptance-based training on empathy toward mental illnesses among nursing students.

**Method** **:** In this clinical trial, 111 nursing students were selected using cluster and quota sampling methods in Mashhad, Iran. They were divided into 3 groups: (1) contact-based education (interpersonal contact among individuals with improved mental illnesses), (2) acceptance and commitment-based training, and (3) control group. The study tool was Jefferson Nurses Empathy Questionnaire, which was completed in 3 stages of pretest, posttest, and follow- up. Data were analyzed by repeated- measures ANOVA.

**Results:** There was no significant difference between contact-based education and acceptance and commitment-based training groups in increasing the average score of total empathy during pretest, posttest, and 1-month follow- up (p = 0/92). However, a significant difference was found between contact-based education and control group (p = 0/004) and between acceptance and commitment-based training and control group (p = 0/02).

**Conclusion: **Both methods of contact-based education and acceptance and commitment-based therapy were effective in increasing the level of empathy into mental illnesses in nursing students.

Nursing education using Bloom's taxonomy of education, is considered as one of the most common educational models in nursing education and involves 3 areas including cognition, emotion, and skillfulness (1). Empathy is considered as one of the most important behavioral abilities in emotional area and can be learned through clinical training (2-4). Also, it can be included in nursing curriculum as a key communication skill (5, 6).

In psychiatric nursing, which is based on communication therapy (7), empathy can reduce internal inferiority perceptions among people with mental disorders by making the patient feel a sense of value, respect, and being accepted (8, 9). It may also be associated with clinical favorable results, such as reducing psychological stresses, improving self-concept, reducing anxiety, depression, and the amount of complaints (10), improving the quality of nursing care, and patient- nurse satisfaction (10,11). 

The review of studies shows that some variables, such as specific clinical experiences, cultural values, and gender, may affect empathy (12). Despite sexual and cultural differences, there are some experiences, such as stress and anxiety of students, which may occur in psychiatric wards before starting psychological clerkship that may reflect students' negative attitude toward psychological disorders (13, 14). Also, moderate or severe levels of stress and anxiety may create more distance between patients and students. Communicating with people who suffer from psychosis, mania, suicide, and anxiety during clerkship may be considered as a barrier in creating effective communication and empathy expression (15).

Social psychologists believe that inter-group contacts under optimum conditions reinforce empathy and reduce prejudice toward other group members by reducing anxiety and other negative emotions more than inter group interactions; and it may also be considered as a starting point for understanding their feelings and attitudes (16, 17). Studying biological basis of mental disorders affects empathy and judgment of clinicians, and it may increase clinicians' empathy by reducing the patients' responsibility for their symptoms (18). So, it seems that contact-based training could improve the level of empathy toward patients by providing an opportunity for contact and interaction between students and patients (17, 19). In this regard, the results of Capozza et al. research (2010) indicates the effects of inter group contacts on reducing anxiety and improving empathy among Italian nurses toward their immigrated coworkers (20). Abd et al. (2015) have also reported that psychiatric nursing training increases the level of empathy toward psychological disorders (21).

Critics of contact-based training hypothesis believe that positive inter group contact only reinforces empathy and reduces prejudice among individuals and it would not be effective in conflict-solving and accepting inter group norms (22). Stereotypes in mental disorders are considered as incompatible schemas that are cognitively inflexible and self-supportive (23). On the other hand, stigma toward mental disorders with roots in language and relationships may be cognitively rigid and self-protective (24). This may lead to stability and continuity in avoidence behaviors due to the dangerous mental illnesses and lack of empathy for them (25). As improving self-acceptance and mindfulness is considered as one of the main personal empathy reinforcement methods toward others (26), it seems that some psychological methods, such as acceptance and commitment training (ACT (which are planned to eliminate the avoidance of difficult feelings and thoughts (27), may reduce stigma and enhance empathy among people who are psychologically inflexible. (26). In this regard, the results of Barbosa et al. (2013) (28) and Milz research (2010) indicated the effect of mindfulness on improving empathy toward patients (29). However, Birnie et al. research indicated contradictory results on students' empathy based on lack of mindfulness effect (30).

Thus, despite the importance of empathy as a valuable tool for communicating with people who suffer from acute psychological disorders, improving empathy in psychiatric nursing training is associated with some challenges including negative attitudes and emotional reactions for psychological disorders (31). Moreover, empathy has been taught by people in the society and strongly depends on socio-cultural conditions (32). On the other hand, empathy toward psychological disorders was also influenced by culture, attitudes, and values (33) and was found to affect the results of researches in this context. Therefore, the limited available studies and lack of generalization of the results of other researches to Iranian students encouraged us to conduct this study to compare the effectiveness of contact-based training and ACT on empathy toward psychological disorders in nursing students.

## Materials and Methods

This clinical trial was conducted on 111 nursing students in their fourth semester in Ibne-Sina Psychiatric hospital in Mashhad- Iran (ethics committee code: 941540 date: 2016/8/17).

**Figure1:**
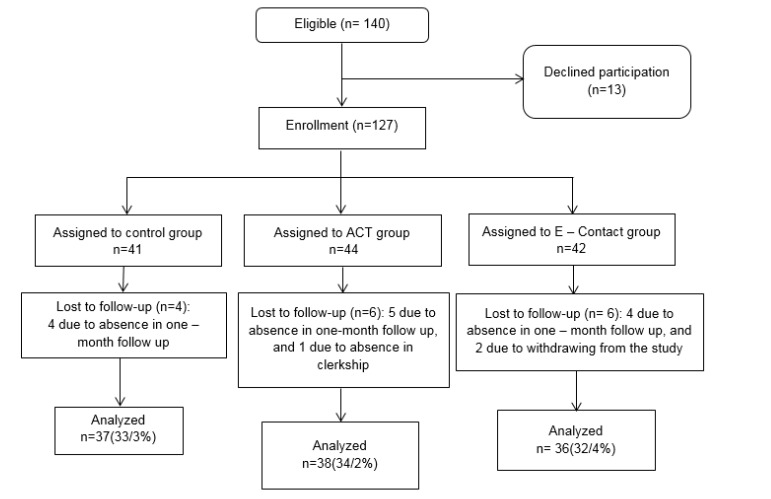
The CONSORT Flow Diagram

According to the pilot study, the sample size consisted of 21 nursing students. They were divided into 3 groups. Based on empathy parameter, 38 individuals were selected for each group and by counting the 15% attrition, 44 individuals were estimated for each group (Figure 1). 

Random cluster and quota sampling methods were used. Nursing faculties training mental health clerkship in Ibne-Sina psychiatric hospital were invited to attend in the study, and accordingly, 12 faculties accepted the invitation, and 4 faculties were randomly selected.

Two groups of male and female students were randomly selected (according to clerkship division group) from each university by quota sampling based on gender distribution. Finally, each group was separately divided into 3 groups of contact-based education, ACT, and control. 

Inclusion criteria were no work experience in psychiatric wards, no psychological disorders, and no mental illness in their first and second degree relatives. Exclusion criteria were reluctance to continue the study, absence of the posttest, and being absent or lack of participation in 1 or more intervention sessions. Teachers' inclusion criteria to the study were holding MA degree in psychiatric nursing or Ph.D. in nursing, having more than 5 years of work experience, and to be a faculty member of the university. 

The study tool was Jefferson Nurses Empathy Questionnaire (34), which contained 20 items and was measured by 7-point Likert scale. It had 3 subscales: (1) adopting empathy viewpoint with 10 items (2, 4, 5, 9, 10, 13, 15, 16, 17, and 20), (2) empathic caring with 7 items (1, 7, 8, 11, 12, 14, 18, and 19), and (3) students empathizing with patients with 2 items (3, 6). Scores ranged from 20 to 140, with higher scores indicating more empathy. Items 11 to 20 were graded in reverse. The questionnaire was translated to Farsi by researchers and was then reviewed by a Ph.D. of English language and a fluent English speaker holding a Ph.D. in clinical psychology. The validity and reliability of the Jefferson Nurses Empathy Questionnaire was confirmed by 10 faculty members of Mashhad University of Medical sciences (CVR = 87%, CVI = 95%), and its consistency was confirmed (α = 92%). 

In contact-based education, 3 patients with improved disorders who were working daily for 4 hours as a connector between different wards of the hospital were selected. They had schizophrenia, bipolar type I, and major depression. The patients were asked to talk about their experiences and personal life with students (Table 1).

According to Steven Hayse protocol (1986), ACT with the content of mental illnesses stigma was held as a workshop by one master of clinical psychology and 2 masters of psychiatric nursing, and the control group received regular clerkship (Table 1).

Both contact-based education and ACT interventions were held in 3 one-hour sessions in the first 3 days of clerkship in addition to the regular clerkship of mental health course 1.

Data were analyzed by SPSS Version 19.5. Chi square and ANOVA test were implemented to investigate the homogeneity of the qualitative and quantitative variables. Repeated- measures ANOVA was used to compare the empathy changes before and after interventions and 1-month after intervention. Statistical significance was considered as 95% confidence interval (CI) level, 85% test ability, and p < 0.05.

## Results

A total of 127 individuals were enrolled in the study and finally 111 individuals were assessed (Figures 1). There were 55% female students and 45% male students, with the mean age of 22.11 years and standard deviation (SD) of 1.56. The 3 groups were homogenous in demographic variables, and no significant differences were found among the 3 groups (Table 2).

According to the results of pre intervention ANOVA, there was no significant difference among the 3 groups in the total score of empathy (p = 0.13) and subscales of adopting empathy viewpoint (p = 0.16), empathic caring (p = 0.15), and students empathizing with the patients (p = 0.30) (table 3).

In inter group comparison, the results of repeated measures ANOVA demonstrated that pretest, posttest, and one-month follow- up stages significantly increased in total empathy mean score and its subscales among the 3 groups of contact-based training, Acceptance and Commitment-Based Training, and control (p<0.05) (table 4). In this regard, the results of post-hoc test for paired comparison with Scheffe correction is presented in Table 5.

## Discussion

The results of the present research showed that empathy mean score increased significantly in both groups of contact-based training and ACT. However, empathy

y mean score in the contact-based education group was higher than the ACT group. On the other hand, both contact-based training and ACT methods were effective in increasing the level of empathy and its subscales in nursing students.

The results of study of Capozza et al. (2010) (20) and Abd et al. (2015) (21) were consistent with those of the present research in the effect of contact and education on empathy improvement. Also, the results of Barbosa et al. (2013) (28) and Mils (2010) were consistent with those of the present study in the effect of mindfulness on empathy improvement (29). The results of Deen et al. research (2010) was based on the effects of stories of people who suffered from psychological disorders on developing empathy among psychiatric residents (35); and it confirmed the results of the present research.

Psychiatric nursing often teaches empathy in some behavioral skills throught listening and speaking. Although teaching empathy is considered valuable in improving interpersonal relationships, it is not sufficient per se (36). Because there are some stressors in clinical environment that would influence theclinical experiences of students and prevent them from applying empathy skills (37). In psychiatric nursing, there are some stereotypes, such as unpredictability and risk of psychological disorders, which prevent facilitation of emotional relationships between patients and nurses by stimulating emotions and negative reactions toward these illnesses and put students in high risk of experiencing clinical stressors (15, 38). 

In contact-based training, positive intergroup interaction leads to new intergroup norms and it would be generalized to new conditions and other members of the group. On the other hand, positive behavioral interactions stimulate greater inter group acceptance from presented anomalies in inter group interactions (39). In contact-based training, listening to experienced distresses, discomforts, and discriminations of patients who suffer from psychological disorders may lead to acceptance of other hospitalized patients who are in acute period of their illness; moreover, it may improve students' ability in understanding patients' attitudes and empathic care by reducing prejudice.

On the other hand, the concept of empathy, which is more related to nursing, is considered as the quality of nurses' presence at the moment (36), and thus, it is necessary to separate the emotional status of nurses and patients (38). In this regard, ACT helps students to experience psychiatric wards as what they really are instead of what the mind creates (41) through encouragement, observation, reducing prejudgment, and empathizing with the patients. 

In fact, positive inter group contact analyzes stereotypes, maintains empathic attitudes toward patients, and disagrees with some attitudes of patients who are a threat to the society (16, 42). The purpose of ACT is to reduce the need to avoid thoughts. So, people are trained to accept thoughts and feelings through cognition of the source of feelings and thoughts with respect to mental illnesses and to reinforce an empathic attitude toward them with no action to reduce them (41).

Birnie et al. (2009) reported that although mindfulness-based training has been effective in reducing personal distress and others' understanding, it is not effective in empathic improvement of students (30); thus, in this sense, it was not consistent with the present research results. Some reasons of this lack of consistency may be the difference between research population and the questionnaire.

**Table1:** Contact-Based Education, ACT, Mental Health Apprenticeship Course 1

**Interventions**	**Sessions**	**Title**	**Content**	**Sessions ** **Implementation**	**Observation and ** **Implementation**
Contact-based education	First session	Patient with schizophrenia	Expressing personal experiences: *About their life and family situations before illness *The way of symptoms detection and their hospitality *Families' behavior with patients after illness *Staffs, nurses and clinicians' behavior during hospitalization *Patients' reaction toward staffs and their expectations *Patients' dreams for future	Interviewing the patient and question and answer between patient and students	Two masters in psychological nursing One clinical master
	Second session	Patient with bipolar 1			
	Third session	Patient with acute depression			
ACT	First session	1.acceptance	Awareness of internal experiences (thoughts, feelings, memories and physical symptoms) in front of mental illnesses and active acceptance of this experiences without any action to reduce it	Workshop	One master in clinical psychology Two masters in psychological nursing
		2.defusion	Not give up thoughts and mental rules related to mental illnesses stigma and find effective interactive methods by the experiences of hospital		
	Second session	3.self as a context	Rooted meaning of stigma from the context of internal events such as thoughts, feelings, memories and physical emotions		
		4.connection with the present time	Effective, open and non- defensive relationship with the present time		
	Third session	5.values	Attention to what is considered as value among people with mental illnesses		
		6.responsibility	Being responsible for behavioral changes into mental illnesses		
Mental health apprenticeship course one	Carried out activities during apprenticeship are as follow: Interview with hospitalized patients, participating in remedial therapy, case report, ECT, training workshops in mental illnesses and relevant treatments training and documentary videos in mental illnesses				

**Table2:** Demographic Features of Participated Nursing Students in Contact-Based Education, ACT and Control Group

**Characteristics**		**E – Contact**	**ACT**	**Control**	**Total**	**Statistics ** **of the ** **Test**
		**n=37**	**n=38**	**n=36**	**n=111**	
		**n (%)**	**n (%)**	**n (%)**	**n (%)**	
gender *	Male	14 (38/9)	16 (42/1)	20 (54/1)	50 (45/0)	Χ^2 ^= 1/90 df=2 P= 0/39
	Female	22 (61/1)	22 (57/9)	17 (45/9)	61(55/0)	
Marital status *	No Married	23 (63/9)	26 (68/4)	30 (81/1)	79(71/2)	Χ^2^ = 2/84 df=2 P= 0/24
	Married	13 (36/1)	12 (31/6)	7 (18/9)	32(28/8)	
*residential area	Urban	33 (91/7)	32 (84/2)	34 (91/9)	99(89/2)	Χ^2^ = 0/77 df=2 P= 0/68
	Rural	3 (8/3)	5 (13/2)	3 (8/1)	11(9/9)	
*faculty of education	Neshapur	7 (19/4)	10(26/3)	7(18/9)	24(21/6)	Χ^2^= 0/86 df=6 p= 0/99
	Gonabad	14 (38/9)	14(36/8)	14(37/8)	42(37/8)	
	Esfaraen	7(19/4)	7(18/4)	8(21/6)	22(19/8)	
	Sabzevar	8(22/2)	7(18/4)	8(21/6)	23(20/7)	
Age**		M (SD)	M (SD)	M (SD)	M(SD)	df=2 F=2/47 P=0/09
		21/64 (1/02)	22/31 ( 2/04)	22/35 (1/36)	22/12 (1/56)	

* Chi-square test statistic

** ANOVA test statistic

**Table3:** Mean and Standard Deviation of Empathy and Its Subscales in Three Studied Groups During Pre-Test, Post-Test and one Month Follow up Session

**Outcome ** **Variables**	**E – Contact group**			**ACT Group**			**Control Group**		
	**T**_3_ **(n=36)**	**T**_2_ **(n=38)**	**T**_1_ **(n=39)**	**T**_3_ **(n=38)**	**T**_2_ **(n=39)**	**T**_1_ **(n=39)**	**T**_3_ **(n=37)**	**T**_2_ **(n=39)**	**T**_1_ **(n=39)**
	**M** **(SD)**	**M** **(SD)**	**M** **(SD)**	**M** **(SD)**	**M** **(SD)**	**M** **(SD)**	**M** **(SD)**	**M** **(SD)**	**M** **(SD)**
View adaptation	50/19 (5/44)	54/64 (5/24)	56/30 (4/98)	51/47 (6/37)	51/71 (6/33)	52/13 (6/20)	48/86 (5/66)	48/11 (5/62)	49/03 (5/38)
Empathic care	41/44 (5/59)	43/55 (4/98)	45/42 (4/59)	41/63 (6/43)	43/31 (6/31)	44/60 (6/01)	39/27 (5/24)	40/16 (5/26)	40/78 (5/03)
Putting themselves instead of patient	11/69 (1/65)	12/64 (1/31)	13/05 (1/07)	11/84 (1/72)	12/47 (1/62)	12/81 (1/37)	11/22 (2/06)	11/32 (2/05)	11/46 (1/94)
Total Empathy	103/33 (11/42)	110/83 (9/93)	114/78 (9/01)	104/95 (13/31)	107/50 (12/96)	109/55 (12/11)	99/35 (11/61)	100/62 (11/48)	101/27 (10/75)

**Table4:** Comparing Mean Changes of Empathy and Its Subscales in Contact-Based Education, ACT and Control Group During Pre-Test, Post-Test and One Month Follow up Sessions

**Outcome ** **Variables**	**E – Contact Group**			**ACT Group**					**Control Group**			**Repeated** **Measures ANOVA** **Test Results** **(Between –** **Subjects Effects)**
	**Standard Deviation(SD)**			**Standard Deviation(SD)**					**Standard Deviation(SD)**			
	**T**_2_ **-T**_3_	**T**_2_ **-T**_3_	**T**_2_ **-T**_3_	**T**_1-_ **T**_2_	**T**_1_ **-T**_3_			**T**_2_ **-T**_3_	**T**_1-_ **T**_2_	**T**_1_ **-T**_3_	**T**_2_ **-T**_3_	
View adaptation	4/44 (2/61)	6/11 (2/94)	1/67 (1/01)	0/24 (1/08)	0/66 (1/47)			0/42 (0/92)	0/24 (0/95)	0/16 (0/96)	0/08 (0/68)	F (2,108) =73/81 P<0/005 Partial Eta=0/58
Repeated measures ANOVA test results (within – subjects effects)	F (2,70) =162/58 P<0/005 Partial Eta=0/82			F (2,74) =8/52 P<0/005 Partial Eta=0/19					F (2,72) =0/45 P=0/64 Partial Eta=0/01			
Empathic care	2/11 (1/33)	3/97 (2/03 )	1/86 (1/24)	1/68 (1/59)	2/97 (2/22)			1/29 (1/16 )	0/89 (0/84)	1/51 (1/22)	0/62 (0/76)	F (2,108) =15/84 P<0/005 Partial Eta=0/23
Repeated measures ANOVA test results (within – subjects effects)	F (2,70) =49/64 P<0/005 Partial Eta=0/59			F (2,74) =26/27 P<0/005 Partial Eta=0/41					F (2,72) =19/53 P<0/005 Partial Eta=0/35			
Putting themselves instead of patient	0/94 (0/98)	1/36 (1/15)	0/42 (0/65)	0/63 (1/12)	0/97 (1/32)	0/34 (0/97)			0/11 (0/46)	0/24 (0/76)	0/13 (0/63)	F (2,108) =7/99 P=0/001 Partial Eta=0/13
Repeated measures ANOVA test results (within – subjects effects)	F (2,70) =43/78 P<0/005 Partial Eta=0/56			F (2,74) =15/62 P<0/005 Partial Eta=0/30					F (2,72) =3/00 P=0/06 Partial Eta=0/08			
Total Empathy	7/50 (3/76)	11/44 (4/95)	3/94 (1/75)	2/55 (3/34)	4/60 (3/17)		2/05 (1/93)		1/27 (1/39)	1/92 (2/14)	0/65 (1/42)	F (2,108) =68/21 P<0/005 Partial Eta=0/56
Repeated measures ANOVA test results (within – subjects effects)	F (2,70) =111/33 P<0/005 Partial Eta=0/76			F (2,74) =4/64 P<0/005 Partial Eta=0/39					F (2,72) =12/38 P<0/005 Partial Eta=0/26			

**Table5:** The Result of Post Hoc Test for Paired Comparison with Bonferroni Correction in Terms of Empathy Mean Changes and Its Subscales During Pre-Test, Post-Test and one Month Follow up

**Scale and subscale**	**Contact- Based ** **Education with ACT**	**Contact- Based ** **Education with Control**	**ACT with Control**
	**P value**	**P value**	**P value**
View adaptation changes	<0/005	<0/005	1/00
Empathic care changes	0/07	<0/005	0/003
Changes of putting themselves instead of patient	0/42	<0/005	0/04
Total changes of empathy	<0/005	<0/005	0/005
View adaptation changes	<0/005	<0/005	1/00

## Limitation

Considering the ethical issues in the present research and due to unavailability of students and due to the fact that some of them were not residents of Mashhad, we could not provide training to the control group.

## Conclusion

Both methods of contact-based training and ACT increased empathy toward patients with mental disorders in nursing students. Therefore, considering the effects of socio-cultural factors on empathy and the need to confirm the results of the present research, more studies should be conducted.
